# Global prevalence of vasovagal syncope: A systematic review and meta-analysis

**DOI:** 10.1016/j.gloepi.2024.100136

**Published:** 2024-01-06

**Authors:** Nader Salari, Zohre Karimi, Mahvan Hemmati, Ali Mohammadi, Shamarina Shohaimi, Masoud Mohammadi

**Affiliations:** a Department of Biostatistics, School of Health, Kermanshah University of Medical Sciences, Kermanshah, Iran; b Student Research Committee, Kermanshah University of Medical Sciences, Kermanshah, Iran; c Department of Biology, Faculty of Science, University Putra Malaysia, Serdang, Selangor, Malaysia; d Cellular and Molecular Research Center, Gerash University of Medical Sciences, Gerash, Iran

**Keywords:** Epidemiology, Prevalence, Reflex syncope, Vasovagal syncope

## Abstract

**Background:**

Today, vasovagal syncope is a common problem that has become a significant health and social challenge. The present study investigated the global prevalence of vasovagal syncope using a systematic review and meta-analysis.

Methods: In this systematic review and meta-analysis study, the global prevalence of vasovagal syncope using the keywords Prevalence, Epidemiology, Vasovagal syncope, and Reflex syncope in PubMed, WoS, Scopus, ScienceDirect databases, and Google scholar search engine without time limit until July 20, 2022, was extracted and transferred to the information management software (EndNote). Then the repeated studies were excluded, and researchers evaluated the remaining studies during three stages (i.e., screening, eligibility, and qualitative assessment). The heterogeneity of studies was investigated using the I^2^ index, and the analysis of eligible studies was performed using the random effects model.

**Results:**

In the review of 12 studies with a sample size of 36,156 people, the global prevalence of vasovagal syncope was reported as 16.4 (95%CI: 6–37.5), and the study of publication bias in the studies through the Egger test shows the absence of publication bias in the studies.

**Conclusion:**

The prevalence reported in the studies shows a high prevalence of vasovagal syncope, which requires serious intervention and preventive, diagnostic, and therapeutic measures. It is necessary for health policymakers to take effective measures in this field.

## Highlights

• The global prevalence of vasovagal syncope was reported as 16.4.
• Egger test shows the absence of publication bias in the studies.
• the I^2^ heterogeneity test showed high heterogeneity (I^2^: 99.7).

## Background

Syncope is a transient loss of consciousness accompanied by a loss of postural tone. Syncope is a common event in the general population and should be considered significant health and social challenge [1]. Vasovagal syncope (VVS) is the most common form of syncope and has a high lifetime cumulative prevalence in the general population [2]. Vasovagal syncope is defined as “the development of hypotension and bradycardia with the typical clinical manifestations of pallor, sweating and weakness” [3]. The complete loss of consciousness in vasovagal syncope usually lasts no more than 20 s [4]. Vasovagal syncope is frequent and benign, and most people do not need special treatment but only reassurance and education [5].

The mechanisms of VVS are not fully understood. There is very little information about the afferent part of the vasovagal reflex (that is, a step from the process to autonomic control and central processing). In contrast, the efferent part of the reflex is quite clear: hypotension and bradycardia. Cardia, respectively, is due to inhibition of the sympathetic system and more or less specific activation of the parasympathetic system [6].

This reflex usually occurs during regional anesthesia, bleeding, or compression of the inferior vena cava in the supine position [7]. Vasovagal-mediated hypotension and bradycardia may severely disrupt cerebral blood flow, and as a result, it causes a sudden and transient loss of consciousness and postural tone [8].

Vasovagal syncope can be classic or non-classic. Classic VVS is caused by emotional or orthostatic stress and can be diagnosed by taking a history. Nonclassical VVS consists of episodes without obvious precipitating events or prodromal symptoms and is diagnosed by partial clinical criteria, exclusion of other causes of syncope, and a positive response to the tilt test (TT). Classic VVS generally begins at an early age. The most common age of onset is 13 years. At the same time, nonclassical VVS often begins at an older age and can be associated with other aberrant disorders such as carotid sinus hypersensitivity, postprandial hypotension, or accompanied progressive orthostatic hypotension [5].

Vasovagal syncope is the most benign type of syncope, with an average prevalence of 22% in the general population. It has a significant medical, social, and economic impact on the general population [7,9]. Approximately 35% of people between 35 and 60 years of age have had at least one episode of VVS. [2]. By age 60, 42% of women and 32% of men experience vasovagal syncope at least once [10]. The risk of vasovagal syncope is approximately between 3% of visits to the emergency room and 5% of outpatient visits [1].

The exact incidence and prevalence of VVS are unknown, and its occurrence may differ among countries due to the variety of exposure to environmental factors and genetic differences [11]. Several studies have described the risk factors of vasovagal reactions. Factors such as young age, weight Low, donating blood for the first time, female gender, hunger, the volume of blood taken, and tachycardia before donating blood can increase the possibility of a vasovagal reaction [12]. VVS is mainly seen in young people and rarely in the elderly. It is more common in women than men, especially when the attack is caused by emotional discomfort. Still, some studies do not consider gender to be effective in the occurrence of this reaction [12,13]. Vasovagal syncope occurs in situations such as fear, pain, prolonged orthostasis, post-exercise reduction of blood supply, and a hot environment [14].

Various studies in the world have reported the prevalence of syncope, these studies have been based on the sample size, year of research, race, and different places, and therefore the reported prevalence's show a high level of heterogeneity. We believe that the use of systematic review and meta-analysis is very useful, in this case, the level of heterogeneity of studies can be reduced and an acceptable prevalence for health policymakers can be presented in the direction of intervention. Therefore, according to the fact that no review study has been done in this area and also according to the differences and heterogeneity of the reported prevalence information the present study aimed to evaluate the global prevalence of vasovagal syncope using systematic review and meta-analysis.

## Methods

In this systematic review and meta-analysis, researchers searched related studies using the keywords Prevalence, Epidemiology, Vasovagal syncope, and Reflex syncope without any time limit until July 20, 2022, among the following databases: PubMed, WoS, Scopus, ScienceDirect, and Google scholar search engine (supplementary). Then, the extracted information was transferred to the information management software (EndNote). Also, the sources used in the identified articles were reviewed manually to maximize the number of related studies.

### Inclusion and exclusion criteria

Study inclusion criteria: 1) Studies that studied the prevalence of vasovagal syncope in different countries. 2) Cross-sectional studies. 3) Studies whose full text was available. 4) Studies that provided sufficient data (sample size and prevalence).

Study exclusion criteria: 1) Conference holding studies 2) Case report studies 3) Case series studies 4) Duplicate studies.

### Selection of studies

Researchers used EndNote software to organize the studies. At first, the studies repeated in different databases were excluded. In the initial evaluation, the titles and abstracts of the articles were accurately examined, and unrelated articles to the research purpose were removed. In the second stage, i.e., the secondary evaluation of the studies, the full texts of the articles were examined, and the studies that met the inclusion criteria were included. 52 articles entered the third stage, which is the qualitative evaluation stage. To avoid the risk of bias and mistakes, all steps of article search, study selection, quality assessment, and data extraction were done by two researchers independently. In case of disagreement between the researchers regarding the inclusion of the article in the study, to avoid the risk of bias for the disputed studies, the reviews were conducted through discussion and with the participation and opinion of the third researcher, and a final agreement was reached.

### Evaluation of the quality of studies

In order to validate and evaluate the quality of articles, a checklist was used according to observational studies. The Strengthening the Reporting of Observational Studies in Epidemiology checklist (STROBE) consists of six scales: title, abstract, introduction, methods, results, and discussion. In total, this instruction consists of 32 items. These 32 items include different methodological aspects of the study, including the title, statement of the problem, study objectives, type of study, the statistical population of the study, sampling method, determining the appropriate sample size, definition of variables and procedures, study data collection tools, statistical analysis methods, and findings. Based on this, articles with a score of 16 and above were considered to be of good and moderate methodological quality, and articles with a score below 16 were considered to be of poor methodological quality and therefore excluded from the study.

### Data extraction

Data extraction was done by two researchers using a previously prepared checklist. This checklist included: the author's name, publication year, research location, sample size, gender and age of participants, data collection tool, and prevalence percentage.

### Statistical analysis

The heterogeneity of the studies was evaluated using the I2 test, and due to the high heterogeneity in this study, the random effect model was used to combine the results of the studies. Publication bias was assessed by Egger's test and corresponding funnel plots were drawn. The data were analyzed in the comprehensive meta-analysis software (version 2) and also used to investigate the effect of the factors affecting the heterogeneity of the meta-regression test for the two factors of the sample size and the year of the study.

## Results

**Fig. 1:**
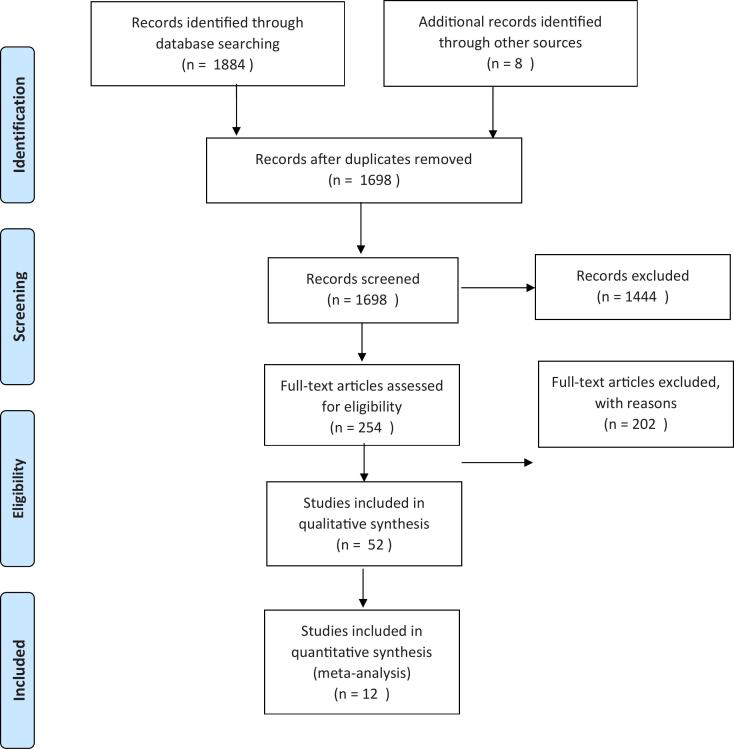
Flow chart indicating the stages of article selection in this systematic review and meta-analysis.

In this systematic review and meta-analysis study, the information of the conducted studies shows the global prevalence of vasovagal syncope. The studies were evaluated based on the PRISMA guidelines. A total of 1884 possible articles from different databases and 8 articles were manually searched and identified. Then, they were transferred to the information management software (EndNote). 194 articles were removed due to duplication. In the primary evaluation stage, 1698 articles were examined, of which 1444 articles were removed based on the inclusion and exclusion criteria, and 254 articles entered the second evaluation stage. In the second evaluation stage, after reviewing the full text of the remaining articles, 202 articles were excluded from the study, and 52 articles entered the qualitative evaluation stage. Of these, 40 articles were removed by checking the checklists, and finally, 12 articles were finalized (Fig. 1), and their information was mentioned in Table 1 (Table 1).

**Table 1:** Information extracted from studies.

Author	Year	Region	Populations	Prevalence	Age	Instruments
V.Casini-Raggi et al. [1]	2002	Italy	500	23%	15–65	Self-expression, physical examination, basal ECG and hematological routine tests
E.Demir et al. [9]	2016	USA	107	22%	14–44	Head-up tilt test, transthoracic echocardiography,24-h monitoring, blood specimens and questionnaire
M.N.Faddis et al. [15]	2002	USA	65	11%	–	Self-expression, physical examination, history, carotid sinus massage, title table testing and ambulatory electrocardiography
I.S.Al-Busaidi et al. [16]	2020	New Zealand	839	42.8%	35–73	Continuous electrocardiogram (ECG)monitoring(telemetry), repeated measurements of lying and standing blood pressure (BP) and if appropriate, withdrawal of offending medication
N.colman et al. [17]	2004	Netherlands	650	29%	60	Medical history, physical examination, 12‑lead electrocardiogram and specific tests
A.Abbasnezhad et al. [12]	2018	Iran	657	2.16%	–	Questionnaire, check list and Sysmax device (K21 Germany)
R.K.Agarwal et al. [18]	2016	India	30,928	2.2%	–	Form, hemoglobin test, view and report
F.Ammirati et al. [8]	1998	Italy	63	42.85%	–	Head-up tilt test, monitoring, history, physical examination, neurological assessments, full routine laboratory tests,12‑lead standard ECG, exercise ECG, Doppler echocardiography, 24-h ECG monitoring, EEG, duplex ultrasound scanning of the carotid arteries, carotid sinus massage, CT scan and MRI
M.Negrusz-Kawecka et al. [11]	2017	Poland	392	1.32%	18–32	Questionnaire and self-statement
M.Brignole et al. [19]	2000	England-USA	189	43%	–	Passive tilt test
M.Ghariq et al. [20]	2020	Netherlands-USA	766	64%	–	Tilt table test (TTT)
M.Pin Tan et al. [21]	2008	England	1000	21.2%	–	Head-up tilt table test(HUT)

**Fig. 2:**
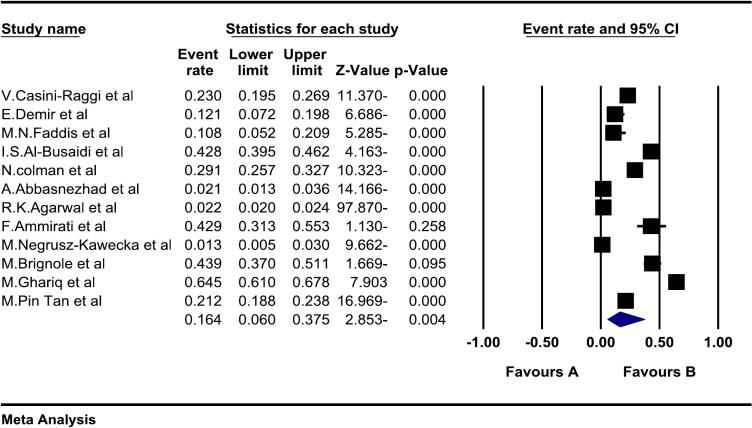
Forest plot of the prevalence of vasovagal syncope based on the random effect's method.

**Fig. 3:**
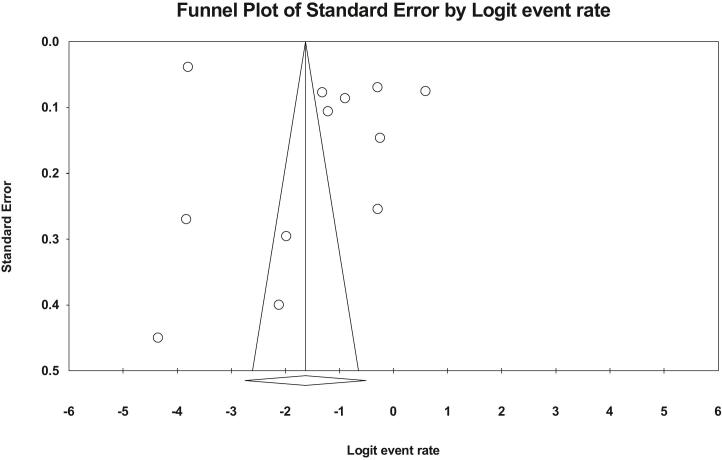
Funnel plot of publication bias in reviewed studies.

In the review of 12 studies with a sample size of 36,156 people, the I^2^ heterogeneity test showed high heterogeneity (I^2^: 99.7), In general, heterogeneity is classified into three categories, low heterogeneity: heterogeneity less than 25%, moderate heterogeneity: between 25 and 75%, and high heterogeneity: more than 75%. and accordingly, Given the high heterogeneity reported across studies, the random effects model was used to analyze the results. Therefore, based on the meta-analysis, the prevalence of vasovagal syncope was 16.4 (95%CI: 6–37.5) reported (Fig. 2), Prevalence is based on the fixed effects method 12 (95%CI: 11.5.12.6), which shows the effect of heterogeneity, so due to the high heterogeneity in this study, the random effects method is desired. and the examination of publication bias in the studies through the Egger test shows the absence of publication bias in the studies (Fig. 3).

**Fig. 4:**
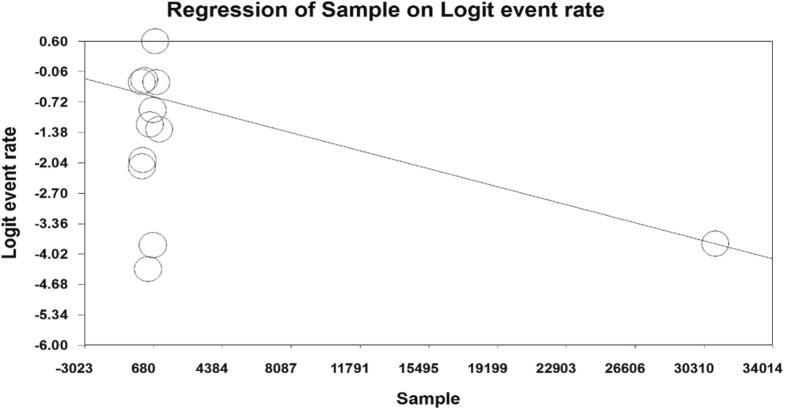
Meta-regression of the effect of sample size on the prevalence of vasovagal syncope.

**Fig. 5:**
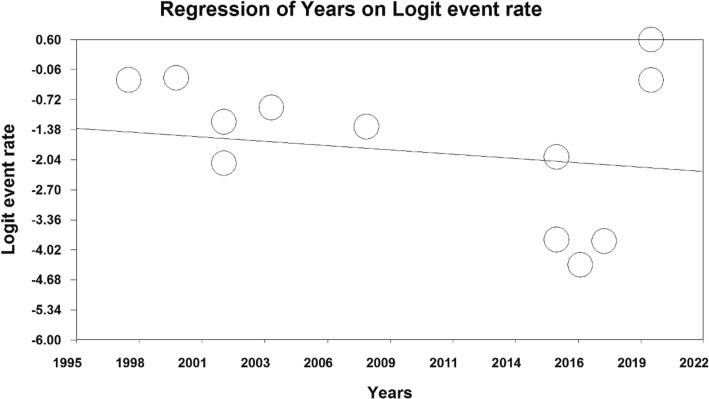
Meta-regression of the effect of the year of studies on the prevalence of vasovagal syncope.

In examining the factors affecting the heterogeneity of studies and examining the effect of sample size on this heterogeneity, it was reported that with the increase in sample size, the prevalence of vasovagal syncope decreases (Fig. 4). Also, with the increase in the years of studies, the prevalence of vasovagal syncope decreases. Also decreases (Fig. 5).

## Discussion

Despite the lifetime prevalence of at least 20–40% of vasovagal syncope, much remains to be learned about it [22]. there are similar proportions of the cumulative incidence of syncope during life in the range of 25–35% in countries such as Canada, the Netherlands, and Malaysia. Therefore, it seems that only a part of people has vasovagal syncope [23].

High heterogeneity was reported in the studies, which is definitely caused by the effects of different sample sizes, different age groups, different races investigated in the studies, different geographical areas, lifestyles, and even the quality of the investigated articles, which according to the information reported in the studies in two In the case of the sample size and the year of conducting the research, meta-regression analysis was performed, considering what was mentioned, this heterogeneity is common, and if future studies are conducted according to a certain age group and the minimum sample size is the same, we can expect that the heterogeneity of the studies will be reduced.

Several factors are involved in the occurrence of vasovagal reactions. The results of our research show the relationship between the incidence of vasovagal seizures with sex, age, weight, blood donation history, feeling of weakness or anxiety before blood donation, BMI, and mean arterial blood pressure [12]. Standing for a long time, changing body position, and hot environments are factors that aggravate VVS. However, older patients are significantly less likely to report the above symptoms. The lack of clear warning signs and symptoms for many elderly patients with VVS who present with transient loss of consciousness and unexplained sudden falls emphasizes the need for specialized evaluation of older patients [24].

Vasovagal syncope (VVS) affects women 1.5 times more than men. The higher rate of vasovagal reaction in women may be mainly related to their lower weight and size [12,20].

Many studies have introduced age as an important factor in vasovagal reactions. With increasing age, the probability of occurrence of vasovagal reactions decreases. Some studies show that the incidence of vasovagal reactions is higher in young people [12]. VVS appears in about 1 to 3% of toddlers as syncope with reflex anoxic syncope or respiratory arrest, and its incidence is significantly higher in It increases around 11 years of age. The average age of the first fainting is about 14 years, and most people with VVS experience their first fainting before age 40 [10]. VVS seems to show a bimodal age distribution with a peak in adolescence and after 60 years of age [20]. Vasovagal syncope in the older population may not follow the benign course usually seen in younger people [21].

The results of our study show that the possibility of vasovagal reaction is higher in people with less weight. Some other studies also confirm this finding [12].

Some clinical features and recent data suggest that unexplained falls and syncope during sleep hours may also be clinical manifestations of VVS. However, VVS in the supine position is rare [13].

Only humans faint. Observations like this suggest that there may be a genetic predisposition to vasovagal syncope, which seems to run in some families. Recently, a large candidate-gene approach of relatives with a high prevalence of vasovagal syncope across multiple generations identified three genes associated with vasovagal syncope. Our understanding of the genetic correlates of vasovagal syncope is in its infancy, and much remains to be understood [23].

Identifying differences in quality of life and psychological well-being between VVS patients and healthy individuals confirm the impact of VVS and promotes greater awareness among health care professionals and the patient population [2]. Our team recently published that the quality of life in patients suffering from VVS is worse than in the general population and similar to patients with New York Heart Association class II-III heart failure. Recurrence in patients with VVS is about 30% [25].

Health interventions as well as policy-making in the field of reduction or prevention and identification and treatment of any disease in society require access to accurate information, when the information of a disease is different and heterogeneous, in practice, policy-making based on this information will not have any useful intervention effects in the society, so the resulting information From this systematic review and meta-analysis, a specific and accurate prevalence can be provided to health policymakers for accurate policy making.

## Conclusion

The prevalence reported in the studies indicates a relatively high prevalence of vasovagal syncope, which requires serious intervention and preventive, diagnostic, and therapeutic measures. It is necessary for health policymakers to take effective measures in this field.

## Funding

Not applicable.

## Ethics approval and consent to participate

Not applicable.

## Consent for publication

Not applicable.

## CRediT authorship contribution statement

**Nader Salari:** Conceptualization. **Zohre Karimi:** Conceptualization, Data curation, Writing – original draft. **Mahvan Hemmati:** Writing – original draft, Writing – review & editing. **Ali Mohammadi:** Data curation, Investigation, Writing – original draft. **Shamarina Shohaimi:** Writing – review & editing. **Masoud Mohammadi:** Conceptualization, Data curation, Formal analysis, Investigation, Methodology, Resources, Software, Supervision, Validation, Writing – original draft, Writing – review & editing.

## Declaration of competing interest

The authors declare that they have no conflict of interest.

## Appendix A Supplementary data

Search strategy Image 1
