# Supplementary material for: Global prevalence of vasovagal syncope: A systematic review and meta-analysis
Source: Glob Epidemiol. 2024 Jan 6;7:100136. doi: 10.1016/j.gloepi.2024.100136 (PMC10821537; doi:10.1016/j.gloepi.2024.100136)
Supplement: Supplementary file 1 — Search strategy [file mmc1.docx]

| Database | Search strategy |
| --- | --- |
| PubMed | (((((Prevalence [MeSH Terms]) AND ("Epidemiology ")) AND ("Vasovagal syncope ")) OR ("Reflex syncope ")) OR ("Syncope, Cerebral ")) OR (Syncope, Malignant Neurocardiogenic) OR ("Syncope, Neurocardiogenic ")) OR (Syncope, Vasovagal, Neurally-Mediated) |
| Web of science | #1: ALL= (Prevalence AND Epidemiology AND " Vasovagal syncope " OR " Reflex syncope " OR " Syncope, Neurocardiogenic " OR " Syncope, Vasovagal, Neurally-Mediated") |
| Scopus | TITLE-ABS-KEY Syncope, Vasovagal, Neurally-Mediated |
| ScienceDirect | Title, abstract or author-specified keywords (Vasovagal syncope OR " Reflex syncope " OR " Syncope, Vasovagal, Neurally-Mediated ") AND ("Prevalence") AND "Epidemiology") |
| Google scholar | (Vasovagal syncope OR " Reflex syncope " OR " Syncope, Vasovagal, Neurally-Mediated ") AND ("Prevalence") AND "Epidemiology") |
